# Vulnerability of Soil Microbiome to Monocropping of Medicinal and Aromatic Plants and Its Restoration Through Intercropping and Organic Amendments

**DOI:** 10.3389/fmicb.2019.02604

**Published:** 2019-11-19

**Authors:** Pooja Misra, Deepamala Maji, Ashutosh Awasthi, Shiv Shanker Pandey, Anju Yadav, Alok Pandey, Dharmendra Saikia, C. S. Vivek Babu, Alok Kalra

**Affiliations:** ^1^Microbial Technology Department, Central Institute of Medicinal and Aromatic Plants, Council of Scientific and Industrial Research, Lucknow, India; ^2^Academy of Scientific and Innovative Research, Central Institute of Medicinal and Aromatic Plants, Council of Scientific and Industrial Research, Lucknow, India; ^3^Analytical Chemistry Department, Central Institute of Medicinal and Aromatic Plants, Council of Scientific and Industrial Research, Lucknow, India; ^4^Department of Molecular Bioprospection, Council of Scientific and Industrial Research, Lucknow, India; ^5^Central Institute of Medicinal and Aromatic Plants, Research Centre, Council of Scientific and Industrial Research, Bengaluru, India

**Keywords:** medicinal and aromatic plants, rhizosphere, microbial richness, microbial diversity, evenness, state shift

## Abstract

Cultivation of medicinal and aromatic plants (MAPs) is persistently increasing due to excessive demands of naturals. Agricultural land and its microbial diversity are primarily adapted to conventional crops, and introduction of MAP and their continuous monocropping may disturb the ecological stability of soil microbiome. Here, the effect of cultivation of MAPs on soil microbial diversity was studied. The aim of the study is to examine the effects of cultivation of MAPs on the possible shift in soil microbial diversity and to restore such impacts by using organic amendments or intercropping. Terminal restriction fragments polymorphism (TRFLP) and next-generation sequencing (NGS) studies showed that of the various selected MAPs, maximal modulation in the soil microbial diversity patterns was noticed in fields of *Mentha arvensis* and *Artemisia annua*, and the traces of essential oil/phytochemicals were detected in bulk and rhizospheric soil. In both *Artemisia*- and *Mentha*-cultivated soil, the total operating taxonomic unit (OTU) declined in both bulk and rhizospheric soil in comparison to control (*Zea mays*), but the bacterial richness of *Mentha* soil was slightly higher than that of control. However, cultivation of *Mentha* improved the evenness of the microbial community. The inclusion of crops like *Sesbania* and *Chlorophytum* and the application of vermicompost (VC) enhanced the microbial richness and evenness, thereby restoring the soil microbial state shift and resulting in higher productivity in the continuously *Mentha* cropped field. Our study concludes that long-term cultivation of some MAPs may affect the richness but promote the evenness of microbial diversity. The state shift could be restored to some extent, and crop productivity could be enhanced by the inclusion of selected crops and organic manures in cropping systems.

## Introduction

The rhizosphere is an active portion of the soil where root influences the processes arbitrated by soil microbiome (Pinton et al., 2007). The composition of root exudates is affected by the interaction of biological, climatic, and physicochemical characteristics of the habitat (Walker et al., 2003). Plant selects certain types of microbes from the rhizospheric diversity through modulating the nature of root exudates and other processes (Wang et al., 2018). Root exudates like sugars, sterols, amino acids, and organic acids affect the pH of rhizospheric soil and solubilize nutrients bound with minerals (Bowsher et al., 2016). This cocktail of chemicals shapes the microbial community within the rhizosphere.

Presence of secondary metabolites and essential oils makes plant the most important source of medicine and drugs (Wink, 2015). With increased global interest toward naturals, there has been considerable interest toward the cultivation of medicinal and aromatic plants (MAPs). In India alone, these crops are expected to cover about 500,000 hectares of farmland in future, and 50,000 tons of essential oil is expected to be produced annually for perfumery, cosmetics, and pharmaceuticals industries generating business of about Rs. 5000 crores. Cultivation of MAPs introduces some novel antimicrobial chemicals in the cropping system, which can negatively affect the indigenous microbial community. It can disturb established microbial networks that have been contributing to crop production and health.

Mints (*Mentha* sp.) are well-known aromatic crops, cultivated for their essential oils, which are commonly used as flavors and stimulants; are carminative in nature besides possessing antimicrobial, antiviral, and insecticidal activities; are quite popular in the Indo-Gangetic plains; and are being cultivated year after year because of their high income-generating potential. Similarly, cultivation of an antimalarial plant *Artemisia annua* has gained importance as *Artemisia* yields artemisinin, an alkaloid recommended by WHO as artemisinin-based combination therapies (ACTs) for the treatment of uncomplicated malaria (Steketee and Thomas, 2017). Introduction of novel crops in a new area can affect the ecological state of pre-existing agro-ecosystems. Many MAPs possess invasive characters, and their wild relatives are strong invaders (Hejda et al., 2009). MAPs can release novel chemical compounds in soil – many of them are antimicrobial in nature – that have never been experienced by soil biodiversity. Such perturbations result in the state shift in soil microbial community, which, in turn, affect the plant–soil feedback and the productivity of agroecosystems. It is expected that their long-term cultivation may affect the diversity and abundance of microbial species because of the antimicrobial impact caused by litter deposition, which may contain a substantial amount of essential oil/aroma chemicals and alkaloids. This can result in major shifts in diversity, which may impact the growth and yield of successive crops.

However, consistent exposure may promote tolerance and adaptation in microbial populations. Such processes may lead to alternative ecological state and evolutionary rescue of microbial communities in the long term. Therefore, the structure and function of soil microbial community will vary with time after the introduction of MAPs. These uncertainties for growing MAPs are great concerns. Moreover, agricultural practices like the addition of the chemical as well as organic fertilizers, crop rotation, mixed and intercropping, etc., can also influence the impact of MAPs on soil microbial diversity. Organic fertilizers including farmyard manure (FYM) and vermicompost (VC) are preferred globally to increase soil fertility, productivity, and sustainability. They are known to improve plant growth by improving enzymatic activity, soil aeration, and maintenance of high microbial populations and activities (Okwuagwu et al., 2003). The introduction of *Sesbania* as green manure in cropping systems increases the yield of the subsequent crops, possibly through the significant improvement of the soil carbon and nitrogen status. Intercrops are known to potentially reduce weeds, diseases, and pests (Trenbath, 1993; Altieri, 1999) and are often regarded as determinant factors influencing crop production (Seran and Brintha, 2010; Steketee and Thomas, 2017).

However, no report is available on the effect of long-term cultivation of MAPs on microbial diversity, which may affect soil health and productivity. This information will help to identify good agricultural practices that could restore the state shift, minimizing loss in soil health and maintaining ecosystem functionality and environmental sustainability. This study was mainly aimed at assessing the possible impact of long-term cultivation of various species of MAPs on the soil microbial diversity. The other objective of the study was to identify the plant species and organic manures that may greatly help the microbial diversity to rescue or resilience and to strategize the inclusion/exclusion of such crops in the MAPs-based cropping system to maintain the microbial richness for higher productivity on sustainable agriculture basis.

## Materials and Methods

A preliminary experiment was designed to analyze the differences in rhizospheric microbial diversity of various medicinal and aromatic crops (*A. annua*, *Andrographis paniculata*, *Mentha arvensis*, *Ocimum basilicum*, *Chrysopogon zizanioides, Pelargonium graveolens*, *Bacopa monnieri*, and *Cymbopogon citratus*) and compared with conventional crop maize (*Zea mays*). Rhizospheric soils of various MAPs cultivated in the demonstration area of CSIR-Central Institute of Medicinal and Aromatic Plants (CSIR-CIMAP) were subjected to Terminal restriction fragments polymorphism (TRFLP). On the basis of more pronounced changes, wide cultivable area under cover, and the higher expected expansion of the crops, *M. arvensis* and *A. annua* were selected among the different MAPs under study (Experiment 1). To further investigate the diversity change at the taxonomic level, bulk and rhizospheric soil of both *Mentha* (MB and MR, respectively) and *Artemisia* (AB and AR, respectively) fields were compared with a conventional crop (*Z. mays*) that served as control (CB and CR), by using the next-generation sequencing (NGS) technique.

The plant–microbe interaction and soil microbial diversity are not only dependent on plant type but also influenced by agricultural practices. To evaluate the effect of various agri-input/agronomical interventions on soil microbial diversity and plant productivity, another study was designed (Experiment 2) involving *Mentha*, which covers a large area, and it was thought appropriate to study its long-term cultivation effects on microbial diversity as about 600,000 farming families are involved in cultivation of this crop on about 300,000 hectares.

### Experimental Plan (Experiment 2)

The experimental design consisted of planting *M. arvensis* in the earthen pots (12″ dia) that were supplemented with chemical (NPK at 37.5, 60, and 50 kg ha^–^1, respectively)/organic fertilizers (VC at 19.76 t ha^–1^ and FYM at 10 t ha^–1^) (Reddy, 2005; Maji et al., 2017) in the month of February (first plantation). VC of the same origin (prepared from distilled waste of lemongrass) has been used for this study. Sterilized VC and FYM were used for all the treatments to minimize the impact/interference of microbiota present in VC and FYM. For the experimental pots where green manures/other crops followed after the first harvest, only basal dose chemical fertilizers (NPK at 37.5, 60, and 50 kg ha^–1^, respectively) were given. The pots receiving chemical/organic fertilizers were maintained with *Mentha* throughout the experiment. The crop was harvested after 90 days in the month of May. The pots were replanted with *Mentha* and/or other crops in rotation and were harvested in August (second plantation). To check the influence of monocropping of *Mentha*, *Mentha* with some rotational crop and MA with ample use of organic fertilizers on its productivity, all the pots were replanted with *Mentha* (third plantation) that were harvested in the month of October (see Supplementary Material for Experimental plan). *Mentha* pots receiving only chemical fertilizer and a non-aromatic crop (maize) were also included in the study, but on the basis of preliminary results (TRFLP), only samples exhibiting prominent changes [*Mentha* (M), *Mentha* + VC (M + VC), *Mentha*_*Sesbania* (M_S), and *Mentha*_*Chlorophytum* (M_C) of the second plantation] were included for the NGS analysis.

Plant weight of above ground herb was recorded and essential oil extraction in the fresh herbage was performed at the time of first and third harvesting by using hydro-distillation in Clevenger’s apparatus (Maji et al., 2013). The oil yield (grams per pot) was calculated by multiplying the amount of oil obtained (ml) with a value of specific gravity (0.89) of *Mentha* oil.

### Soil Samples

The soil samples were collected in triplicate from the fields of CSIR-CIMAP continuously grown with MAPs, i.e., *A. annua*, *A. paniculata*, *M. arvensis*, *O. basilicum*, *C. zizanioides*, *P. graveolens*, *B. monnieri*, and *C. citratus*. To ensure that change in rhizospheric microbial diversity is a result of the cultivation of the selected crops, soil samples were taken from the field just before the harvesting of crops. The rhizospheric soil (soil firmly adhering to the root) was collected and stored at −20°C for further experimentations. To reduce the sample to sample variation, three to five samples from each of the three plots were drawn and mixed to form a plot sample and three such plot samples were mixed to form a composite sample for each treatment for NGS analysis.

### TRFLP (Terminal Restriction Fragment Length Polymorphism)

Total DNA extraction was done by using the Power Soil DNA Extraction Kit (Mo Bio Laboratories) according to the manufacturer’s instructions. Isolated DNA from soil samples was checked qualitatively and quantitatively on 0.8% agarose gel electrophoresis and Nanodrop yield, respectively. PCR was performed using a standard protocol (Bharti et al., 2015). PCR mixtures containing 25 ml of Premix Taq (Takara Biotechnologies), 2 ml of the DNA template, and 1.5 ml of each primer were made up to a volume of 50 ml with sterilized Milli-Q water. The samples were amplified in an Eppendorf Thermal Cycler. Tetra base cutter restriction enzymes *Alu*I and *Hha*I (Promega) were used to digest the fluorescently labeled PCR amplicons.

### T-RFLP Analysis

For TRFLP, 1 μl of the restriction-digested product was mixed with 12 μl of freshly deionized formamide containing 0.5 μl of LIZ 500 marker (PE Applied Biosystems). 3130xl Genetic Analyzer (Applied Biosystems, United States) of Gene Scan mode (Moeseneder et al., 1999) was used for detection as well as for the separation of DNA fragments. Raw data from Gene MapperTM were exported to T-REX, online software for the processing and analysis of T-RFLP data^1^ (Culman et al., 2009). Raw files were analyzed by the Past 2.02 software package (Hammer et al., 2001).

### Next-Generation Sequencing

Soil samples from experiment 1 were outsourced to Genotypic Technology Pvt. Ltd., and soil samples from experiment 2 were outsourced to Bionvid Technology Pvt. Ltd., for metagenomic studies. Samples outsourced were processed and sequenced by using Illumina MiSeq as per sequencer manufacturer’s protocol. Sequence data have been deposited in the NCBI Sequence Read Archive (SRA) database. The sequences were submitted to NCBI, and the accession numbers obtained are SUB2488937 and SUB2604678 for 16S and SUB2604678 and SUB2659621 for ITS for experiment 1 and SRP150923 for 16S and SRP150978 for ITS for experiment 2.

### Statistical Analysis for Metagenomic Sequencing

The dataset was analyzed using Qiime2 v 1.9.0 by using the Greengenes3 v 13.8 database at the backend. The dataset was clustered at 97% similarity and mapped to the reference using the UCLUST ref method. Abundance graphs were plotted based on the number of hits. Comparative taxa summary plots and heatmap were generated across the samples at phyla level considering relative abundance (RA) values using a cutoff greater than 0.1%. Krona charts were plotted using Krona5 tools for each sample. Pie charts were generated for each sample from phylum to species with a cutoff of 0.5% based on the absolute abundance count. Alpha diversity was calculated using Chao1, observed operating taxonomic units (OTUs), Shannon, and Simpson index. All the data and graphs were analyzed by R software (Vegan and ggPlot packages), SPSS 16.0, and PAST 2.02 (details in Supplementary Material).

### GC-MS/HPLC of Soil Samples of the Selected Crops

Five grams of soil samples are used for analysis to check the signatures of essential oil/artemisinin in bulk and rhizosphere soil samples of maize, *Mentha*, and *Artemisia* using standard protocols (Gupta et al., 2002; Dundek et al., 2014).

### Soil Samples and Their Physiochemical Study

The pH of collected soil samples was determined using Equiptronic’s pH meter as described by Jackson (1967). The electrical conductivity (EC) of a soil sample was determined by Equiptronic‘s digital EC bridge. Nitrogen (N) in the soil is mainly present in organic form together with small quantities of ammonium and nitrate ions and was determined by distilling soil with alkaline potassium permanganate according to Subbiah and Asija (1956). Soil-available phosphorus (P) is found as orthophosphate and was extracted by Olsen’s extract 0.5 M NaHCO_3_ (pH 8.5) (Olsen et al., 1954) and measured calorimetrically by the ascorbic acid method (Murphy and Riley, 1962). The ammonium acetate method followed by flame photometric detection (Hanway and Heidel, 1952) was employed to estimate the available potassium (K) of samples. Organic carbon (OC) of all the soil samples was measured using the chromic acid titration method proposed by Walkey and Black (1934).

### Statistical Analysis

All statistical analyses [one-way ANOVA and Correspondence Canonical Analysis (CCA)] for physicochemical properties of soil, plant biomass, and oil yield were carried out using SPSS 16.0, PAST 2.02.

## Results

### Experiment 1

#### Screening of MAPs

Cultivation of MAPs, as shown by the preliminary TRFLP experiments, reflected marked changes in total rhizospheric microbial diversity. The change reflected in the results showed that though some MAPs, like *Chrysopogon*, increased the number of TRFs in the soil over the native soil microbial diversity, some other important MAPs resulted in a decline in microbial diversity. The decline in the microbial diversity was noticed in plots cultivated with *Artemisia*, *Andrographis*, *Mentha*, *Ocimum*, *Pelargonium*, *Bacopa*, and *Cymbopogon*; the decline being maximum in *Mentha* and *Artemisia* (Supplementary Table S1).

#### Diversity, Richness, and Dominance

Higher values of Shannon, Simpson, and Fisher’s Alpha indicating higher richness and dominance, as well as alpha diversity, were noticed in *Pelargonium* and *Chrysopogon* cultivated soils (Supplementary Table S1). Although the values of Simpson and Shannon in soil of other MAPs were lower than maize, maximum decline was observed in *Mentha* (Fisher’s alpha value, 7.542 for bacterial community and 5.916 for fungal community), followed by *Artemisia* (Fisher’s alpha value, 10.76 for bacterial community and 6.308 for fungal community), while an increase in total diversity was noticed in *Chrysopogon* soil (Fisher’s alpha value, 61.48 for bacterial community and 23.55 for fungal community).

To substantiate the role of essential oils/aroma chemicals/alkaloids affecting the microbial diversity, and to detect the presence of the essential oils/artemisinin in the soil, samples were collected after the harvest of the crops and were subjected to GC-MS/HPLC analysis. Limonene, menthyl acetate, and menthol were present in both the bulk and rhizospheric soil of *Mentha*, while neomenthol could be detected in bulk soil only (Supplementary Figure S1a). Relatively more components, as well as the concentrations of aroma chemicals, were detected in bulk than rhizospheric soil. Similarly, the signatures of artemisinin were detected in the AR as well as the AB soil (Supplementary Figures S1c,d) and the amount of artemisinin was higher in AB (0.25 ± 0.01 ppm) than in AR (0.16 ± 0.004 ppm) (Supplementary Table S2). No such signatures were recorded from soil cultivated with maize in both bulk and rhizosphere (Supplementary Figures S1b–d).

#### Physiochemical Parameters of Bulk and Rhizospheric Soil (*Mentha*, *Artemisia*, and Control)

As shown in Supplementary Table S3, the changes in the pH value for both bulk and rhizospheric soils showed fluctuation with an upward trend in all the samples (control, *Mentha*, and *Artemisia*) from bulk to rhizosphere, and EC showed a similar downward pattern in both *Mentha* and *Artemisia* but upward in control soil from bulk to the rhizosphere. Available N and P contents decreased significantly in bulk than the rhizospheric soil in both *Mentha* and *Artemisia* as well as in control. Nevertheless, there was no significant difference in K content of AR, MB, and MR, which were significantly different from AB and control soil (CB and CR). OC content of control bulk and rhizospheric soils was found to be different from Mentha and Artemisia (bulk and rhizosphere).

#### Soil Diversity Analysis Using the NGS Technique

Though the TRFLP experiment clearly indicated a loss in diversity in most of the MAPs, the maximum loss in soil microbial diversity was noticed in *Mentha* and *Artemisia* samples, and the GC-MS and HPLC experiments confirmed the signature/traces of oil and artemisinin, respectively, which may be the reason for these variations. To gain insight into the actual changes occurring in the rhizosphere and bulk soil, and to identify the species deleted, introduced, or shifted because of cultivation of MAPs, soil samples of *Mentha* and *Artemisia* were subjected to NGS using Illumina as the sequencing platform. Taxonomic assignments of the OTUs were done at phylum, class, order, family, genus, and species level for 16S/ITS sequences. Bacterial and fungal community richness and diversity are shown in Table 1.

**TABLE 1:** Diversity indices and OTUs obtained by NGS analysis of bacterial 16S and fungal ITS sequences from bulk and rhizospheric soil of *Mentha arvensis*, *Artemisia annua*, and *Zea mays* (control).

**S.N.**	**Soil samples**	**Shannon**		**Simpson**		**Chao1**		**OTUs**		
					
		**16S**	**ITS**	**16S**	**ITS**	**16S**	**ITS**	**16S**	**ITS**	**Total OTUs**
1.	AB	9.951	7.775	0.997	0.983	6528.023	3199.964	5241	2717	7958
2.	AR	9.963	8.087	0.997	0.987	7738.334	3951.025	6313	3538	9851
3.	MB	9.916	7.087	0.996	0.975	9734.272	2943.519	8336	2364	10,700
4.	MR	9.909	6.061	0.996	0.938	8409.02	6379.28	6913	5541	12,454
5.	CB	9.887	7.587	0.996	0.965	8739.34	6050.786	7164	4375	11,539
6.	CR	10.061	7.786	0.997	0.972	8250.056	12, 996.053	6529	12,566	19,095

*AB = *Artemisia* bulk soil, AR = *Artemisia* rhizosphere soil, MB = *Mentha* bulk soil, MR = *Mentha* rhizosphere soil, CB = control bulk soil, CR = control rhizosphere.*

In the case of a bacterial community, differences in alpha diversity were not prominent in bulk soil but in rhizospheric soil of both plants (*Mentha* and *Artemisia*) and values slightly differed in comparison to maize. The number of OTUs for the bacterial community in AB (5241) was considerably lesser than CB (7164); however, in the case of MB (8336), a higher number of OTUs was recorded. On the other hand, OTUs of the fungal community in bulk soil of AB (2717) and MB (2364) were considerably lower than CB (4375). Similarly, for the bacterial community, in rhizospheric soil, both AR (6313) had lower OTUs in comparison to CR (6529), but MR (6913) had a slightly higher number of OTUs and the difference was not well defined; however, marked differences were noticed in the case of the fungal community; the number of OTUs in AR (3538) and MR (5541) was substantially lower than that in CR (12,566).

#### The Composition of Bacterial Communities

**FIGURE 1:**
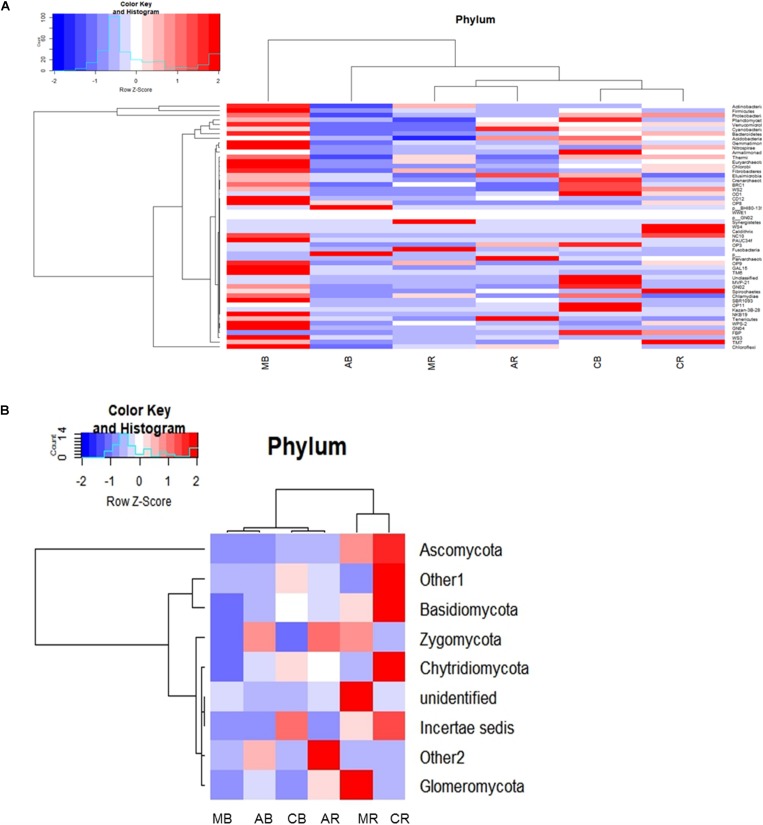
Heatmap representing the relative abundance at the phylum level **(A)** for bacterial and **(B)** for fungal community. AB = *Artemisia* bulk, AR = *Artemisia* rhizosphere, MB = *Mentha* bulk, MR = *Mentha* rhizosphere, CB = Control bulk, CR = Control rhizosphere.

Different soils comprised a diverse number of identified phyla, class, order, family, genus, and species. The RA (represented in heatmap) of the assigned phyla across the (*Mentha* and *Artemisia* soil) samples was compared with control (Figure 1A). The dominant classified bacterial phyla obtained were from Proteobacteria (24–39%), Actinobacteria (16–36%), Firmicutes (9–26%), Chloroflexi (8–14%), Acidobacteria (3–11%), Bacteroidetes (3–6%), Planctomycetes (0.7–5%), Verrucomicrobia (2–3%), Cyanobacteria (1–8%), and Armatimonadetes (1–2%) (Supplementary Figure S2a); the heatmap representing their RA was also generated (Supplementary Figure S2c). The 95–97% of total bacterial OTUs with RA of >1% were Proteobacteria, Actinobacteria, Firmicutes, Chloroflexi, Acidobacteria, Bacteroidetes, Planctomycetes, Verrucomicrobia, TM7, Cyanobacteria, and Armatimonadetes. In addition, Gemmatimonadetes, Nitrospirae, Elusimicrobia, Fibrobacteres, Chlorobi, Euryarchaeota, Creanarchaeota, Tenericutes, OD1, FBP, BRC1, Thermi, WS3, Chlamydiae, and OP11 were present in most samples but at relatively low abundances (RA 0.1–1%), as well as 21 other rarer phyla (RA < 0.01%).

#### The Composition of Fungal Communities

In the fungal community, three dominant phyla (Ascomycota, Basidiomycota, and Zygomycota) were observed that accounted for 86% of the total fungal sequences obtained. The RA (represented in heatmap) of the assigned phyla across the (*Mentha* and *Artemisia* soil) samples was compared with control (Figure 1B). The dominant classified phyla were Ascomycota (28–79%), Basidiomycota (9–23%), Zygomycota (1–15%), Chytridiomycota (0.4–2%), and Glomeromycota (0.1–2%) (Supplementary Figure S2b); the heatmap representing their RA was also generated (Supplementary Figure S2d). The most abundant phyla in case of the fungal community were Ascomycota, Basidiomycota, Zygomycota, Chytridiomycota, and Glomeromycota with a RA of >1%; some unclassified phyla with RA < 1% were also noticed across all the samples (Figure 1B).

The long-term cultivation of *Mentha* and *Artemisia* resulted in a change in the abundance of microbes. The bacterial community abundance pattern in control soils (in both bulk and rhizosphere) showed a similar pattern with a higher abundance of Proteobacteria followed by Actinobacteria and Firmicutes. In the case of *Mentha*, an abundance of bacterial OTUs differed in MB and MR soil. Although the abundance of Firmicutes was found to be higher, which superseded the abundance of Proteobacteria, Actinobacteria, Chloroflexi, Bacteroidetes, and Verrucomicrobia in MB soil, the abundance of Acidobacteria, Planctomycetes, Armatimonadetes, and Cyanobacteria was lower as compared to CB soil. In MR soil, higher abundance of Firmicutes, Actinobacteria, and Chloroflexi was recorded, whereas Proteobacteria, Acidobacteria, Bacteroidetes, Planctomycetes, Verrucomicrobia, and Armatimonadetes were less abundant in comparison to CR soil. In the case of the fungal community of MB soil, lesser abundance of Basidiomycota, Ascomycota, and Chytridiomycota members was found as compared to CB soil. In MR soil, the same pattern was recorded with higher abundance of Zygomycota (Supplementary Figure S2b).

In *Artemisia* soil, the AB bacterial community included Proteobacteria, Firmicutes, Acidobacteria, Actinobacteria, Chloroflexi, Planctomycetes, Bacteroidetes, Cyanobacteria, Verrucomicrobia, and Armatimonadetes, but was less abundant than CB. However, in AR, Chloroflexi, Acidobacteria, Cyanobacteria, and Planctomycetes were more abundant than CR. In the fungal community, abundance of Ascomycota and Basidiomycota were less in AB than CB, but Zygomycota, Chytridiomycota, and Glomeromycota were more abundant. AR had a lower abundance of Ascomycota, Basidiomycota, and Chytridiomycota but a higher abundance of Zygomycota and Glomeromycota in comparison to CR soil (Supplementary Figure S2b).

**FIGURE 2:**
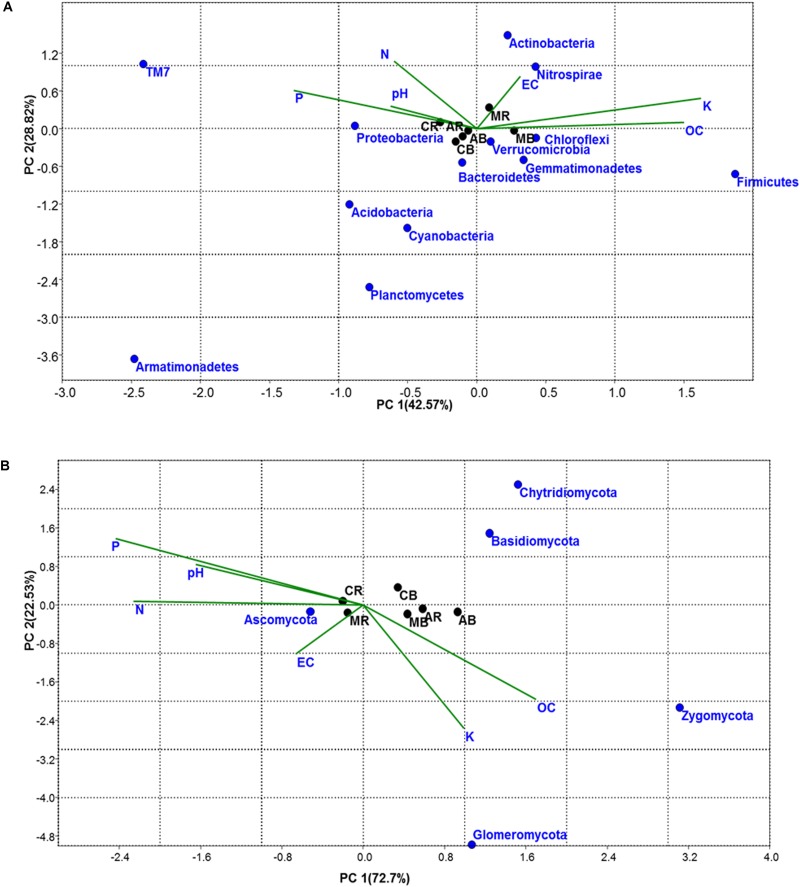
Correspondence Canonical Analysis (CCA) based on OTUs and physiochemical parameters **(A)** for bacterial and **(B)** for fungal community; *P*-value > 1. Similarities and differences in microbial structure and their correlation with physiochemical parameters were carried out using CCA. AB = *Artemisia* bulk, AR = *Artemisia* rhizosphere, MB = *Mentha* bulk, MR = *Mentha* rhizosphere, CB = Control bulk, CR = Control rhizosphere.

Similarities and differences in bacterial/fungal community structure and their correlation with physicochemical parameters were carried out using CCA (Figure 2). CCA showed that the first PC explained 55.38 and 77.13% of the total bacterial and fungal variation, respectively. For bacteria, the first component (PC1), which explained 55.38% of the total variation, separated the MA- and AA-associated soil samples from that of maize (Figure 2A). As shown by their close groupings and vectors, the dominant bacterial phyla in MA-associated soil were Actinobacteria (MR), Firmicutes (MB), Verrucomicrobia (AR), and Chloroflexi (AB), which were found to be affected by pH, EC, N, and OC content. The dominant bacterial phyla in maize-associated soil samples were Proteobacteria and Bacteroidetes. In the case of fungi, the first component (PC1), which explained 77.13% of total variation of fungal phyla, separates rhizosphere from bulk soil samples (Figure 2B). The grouping and vectors of the plot showed that the top fungal phylum in all treatment was Ascomycota and was found to be affected by N, P, pH, and EC.

### Experiment 2

#### Distribution, Richness, and Dominance Based on TRFLP Analysis

To evaluate the effect of various agricultural practices on soil microbial diversity, organics like VC and FYM (major organics used by farmers) were applied to the soil. The physicochemical parameters of the soil samples from first, second, and third harvesting were recorded and are represented in Supplementary Table S4. After the harvesting of the first plantation, *Mentha* soils supplemented with VC showed higher microbial diversity and richness among the other treatments in relation to both bacterial and fungal communities (Supplementary Table S5). In case of bacterial community, the values of Simpson varied from 0.7747 to 0.9075 and those of Shannon varied from 1.992 to 3.043, and for the fungal community, Simpson values ranged from 0.7175 to 0.9348 and Shannon values ranged from 2.153 to 3.268.

The application of VC increased the total richness in terms of the increased value of Margalef (7.738 for bacterial and 5.211 for fungal diversity) and Fisher’s alpha diversity (16.21 for bacterial and 10.7 for the fungal community) in comparison to soil samples with only *Mentha* (without any organic manure). RA (Heatmap) also indicated the shifting of some specific bands in M + VC samples.

The addition of all the organic inputs led to an increment in the soil microbial diversity. The enhancement in the bacterial diversity was maximum when *Sesbania* was included in crop rotation after *Mentha*, followed by the application of VC, whereas in the case of fungal diversity, plantation of *Chlorophytum* after *Mentha* maximally increased diversity, which was closely followed by the application of VC.

In the third plantation, *Mentha* was replanted in all the pots, and the rhizospheric soil samples were subjected to TRFLP. In the case of the bacterial community, M + VC and M_S_M, and in fungal community, M_S_M and M_C_M maximally restored the loss in soil microbial diversity. Values of Simpson (0.8394), Shannon (2.382), Margalef (4.907), and Fisher’s alpha diversity (8.412) were found to be highest in M_S_M in the bacterial community, whereas M_C_M had a maximum value of Simpson (0.9358), Shannon (3.229), Margalef (8.493), and Fisher’s alpha diversity (18.71) in the case of the fungal community (Supplementary Table S5).

#### Total Structural Variance in Microbial Diversity

The coverage of all the soil samples was more than 96%, which showed that the depth of sequencing met the needs of our experiment. The bacterial community was restored to the maximum extent in the treatments M + VC and M_S, where the number of OTUs was brought back from 230 (M) to 244 (M + VC) and 234 (M_S); for the fungal community, M + VC was successful in restoring the diversity loss to the maximum extent [OTUs were brought from 427 (M) to 448 (M + VC)]. In context to diversity indices, a higher value of richness in terms of Shannon index (4.12 for bacterial and 3.47 for the fungal community) and minimum dominance (0.43 for the bacterial community and 0.051 for the fungal community) was recorded in M_C (Table 2).

**TABLE 2:** Alpha diversity and OTUs obtained by NGS of bacterial 16S and fungal ITS sequences from restoration experiment of second plantation.

	**Soil samples**	**OTUs**		**Shannon**		**Simpson Inv mean**		**Chao1**	
					
		**16S**	**ITS**	**16S**	**ITS**	**16S**	**ITS**	**16S**	**ITS**
1.	M	230	427	4.1	3.41	23.34	17.83	147.81	74.9
2.	M + VC	244	448	3.95	3.01	21.7	11.4	146.4	64.99
3.	M_S	234	365	4.03	3.2	23.28	13.87	147.32	69.53
4.	M_C	228	313	4.12	3.47	23.22	19.53	148	77

*M = *Mentha arvensis*, VC = vermicompost, S = *Sesbania*, C = *Chlorophytum*.*

**FIGURE 3:**
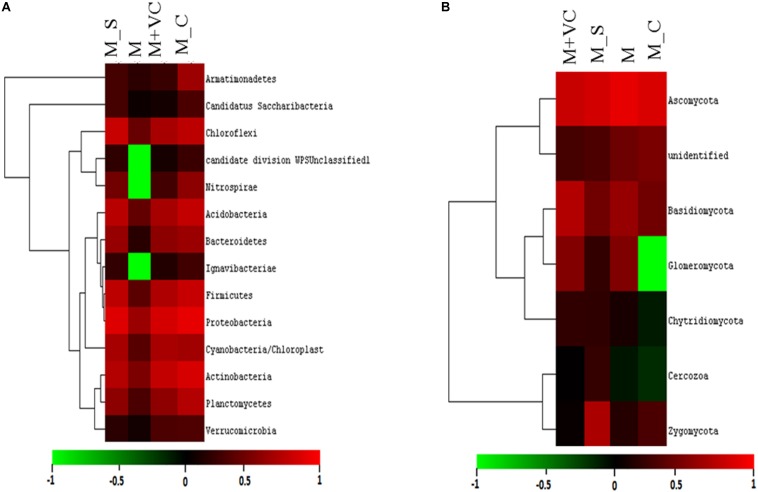
Relative abundance (heatmap) of **(A)** bacterial and **(B)** fungal community after second harvesting soil. M = *Mentha*, M + VC = *Mentha* with vermicompost, M_S = *Mentha* before *Sesbania*, M_C = *Mentha* before *Chlorophytum*.

**FIGURE 4:**
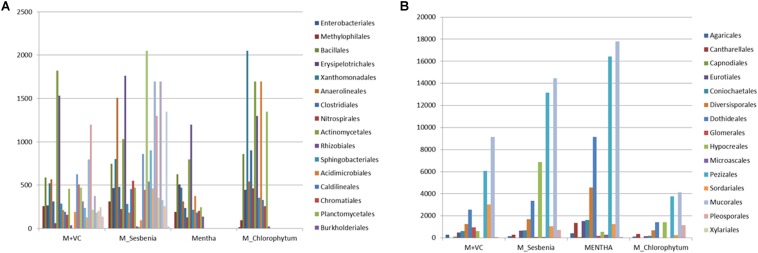
Community rescue profile of **(A)** bacterial and **(B)** fungal communities after application of organic manures and intercropping with *Sesbania* and *Chlorophytum*. M = *Mentha*, M + VC = *Mentha* with vermicompost, M_S = *Mentha* before *Sesbania*, M_C = *Mentha* before *Chlorophytum.*

On the basis of total abundance at the phylum level (Supplementary Figure S3a), an abundance of Proteobacteria (29.18%), Bacteroidetes (4.92%), and Acidobacteria (12.82%) was higher when VC was applied to *Mentha* (M + VC) and in pots where *Sesbania* was planted after *Mentha* (M_S). RA of all the bacterial and fungal phyla are shown in heatmap (Figures 3A,B). In the case of the fungal community, a respective reduction in the abundance of Ascomycetes (46.20 and 54.79%) and Basidiomycetes (4.69 and 6.3%) was observed in both M + VC and M_S, and abundance of Glomeromycetes (17.86%) was higher in *Mentha* supplemented with VC (M + VC) (Supplementary Figure S3b). At the order level, some groups were found to be higher in M + VC, M_S, and M_C as compared to M, but the use of M+CF negatively affected the soil microbial abundance. The bacterial community showing higher resilience or those having being rescued at the order level after the application of VC and organic and green manure were Planctomycetales, Rhizobiales, Actinomyceteales, Anaerolineales, Clostridiales, Xanthomonadales, Acidomicrobiales, Chromatiales, Nitrospirales, Methylophilales, Caldilineales, and Bacillales (Figure 4A). On the other hand, in the fungal community, abundance of Mucorales, Pezizales, Sordariales, Dothideales, and Divesisporales was found to be highest in M-grown soils; only Hypocreales could be rescued in M_S-grown soil (Figure 4B).

**FIGURE 5:**
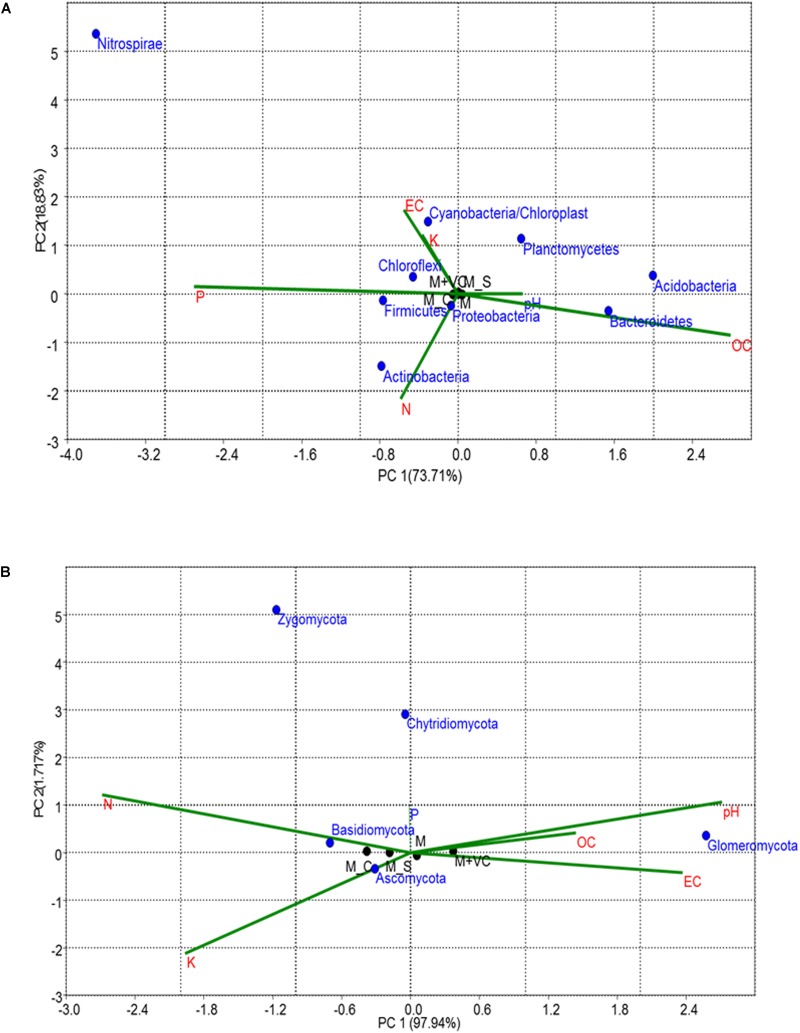
CCA based on OTUs and physiochemical parameters for **(A)** bacterial and **(B)** fungal community; *P*-value > 1. Similarities and differences in microbial structure and their correlation with physiochemical parameters were carried out using CCA. M = *Mentha*, M + VC = *Mentha* with vermicompost, M_S = *Mentha* before *Sesbania*, M_C = *Mentha* before *Chlorophytum*.

Correspondence Canonical Analysis was used to study the similarities and differences in bacterial as well as fungal community distribution along with their correlation with physicochemical soil properties (Figure 5). Significant correlations were found in both bacterial and fungal communities; a total variation of PC 92.54% was observed in the bacterial community and 99.65% in the case of the fungal community (Figure 5A). In the case of the bacterial community, the first principal component (73.71%) separated the M_C and M + VC from other samples. According to their grouping and vectors, the dominant bacterial phylum was Proteobacteria, which was found to be related to N.

On the other hand, in the case of fungi, the first component (PC1) explained 97.94% of the total variation in fungal community distribution, which separated the M_S and M_C from other samples. On the basis of grouping and vectors, the dominant fungal phylum was Ascomycota and was found to be closely related to K (Figure 5B).

#### Plant Biomass and Oil Yield

In the first plantation, harvesting of *Mentha* yielded significantly higher plant biomass in terms of both fresh weight (96.04 g) and oil yield (0.722 g yield per pot), when *Mentha* was supplemented with VC (M + VC) (Supplementary Table S6) while *Mentha* not supplemented with any kind of fertilizer yielded 75.82 g of herb and 0.61 g of oil. During the harvesting of *Mentha* after the third plantation, maximum yield of fresh biomass and essential oil could be achieved when *Mentha* was rotated with *Sesbania* (M_S_M) followed by *Mentha*, which was continuously supplemented with vermicompost (M + VC) and *Mentha* rotated with *Chlorophytum* (M_C_M); the values were 90.50, 75.14, and 65.71 g for fresh biomass and 0.759, 0.624, and 0.580 g per pot for oil yield, respectively (Supplementary Table S7), which indicated that fertility and productivity of *Mentha*-grown soil could be restored in treatments involving *Sesbania*, VC, and *Chlorophytum*.

## Discussion

Medicinal and aromatic plants are grown for essential oils and several phytopharmaceutically important compounds known for their antimicrobial properties. Our study clearly indicates that changes/differences in the microbial commune structure are brought about by monocropping of several MAPs. This could be a result of the cross-talk between some factors including the presence of secondary metabolites released in the soil from defoliated leaves in soil. Though the nature of the response in both bacterial and fungal community was somewhat similar (both the communities affected by MAPs), the extent of response varied greatly. The initial leaf litter decomposition is carried out by the fungal community because they have the ability to break the plant organic matter, but as the succession takes place, the bacterial community gets involved in the decomposition process and help the fungal community to solubilize the complex organic matter (Llado et al., 2017).

### Soil Diversity Changes Brought About by Cultivation of MAPs and Conventional Crops

Plant residues are an important and decisive source of nutrients for the microbial community (Dilly et al., 2000; Schloter et al., 2003). However, the presence of some unique and structurally diverse bioactive molecules called secondary metabolites causes MAPs to harbor a distinctive and specific microbiome (Wu et al., 2015). Reduction in the total soil microbial diversity of *Mentha* and *Artemisia* as compared to maize was reflected by the diversity analysis, which could be attributed to the presence of such bioactive compounds with antimicrobial properties as revealed by GC-MS/HPTLC studies of soil. These bioactive volatile compounds may act as phytoncides for some group of microbes, but the community shift stabilizes the community structure by maintaining the evenness. Components of essential oil in this study, which were found in rhizospheric and bulk soil are known for their antimicrobial properties. While menthyl acetate and menthol are relatively more effective against the bacteria, neomenthol and limonene have been found to be more effective against fungi (Sokovic et al., 2008; Kuriata-Adamusiak et al., 2012; Vieira-Brock et al., 2017). The presence of limonene in both bulk and rhizospheric soil could have played a role in affecting fungal diversity, whereas the greater reduction of fungal diversity in bulk soil might be because of the presence of neomenthol, which was not detected in the rhizospheric soil. In case of bacterial diversity, the presence of menthol and menthyl acetate in both the soils (bulk and rhizosphere) could have resulted in greater reduction in bacterial diversity as compared to the control where no such peaks were detected.

Artemisinin mainly found in trichomes of leaves (Muranaka and Saito, 2010) and excessive leaf fall may release more of this compound in the soil post litter decomposition and its antimicrobial property might have affected both the bacterial and fungal diversity, which was more profound in bulk soil than in rhizospheric soil (Tajehmiri et al., 2014), which again could be correlated with the presence of relatively higher amounts in bulk soil. Moreover, cultivated plant type has a profound influence in determining the microbial species composition of the rhizosphere resulting in distinct community structures for different plant species grown in the same soil (Grayston et al., 1998; Miethling et al., 2000; Patkowska and Konopiński, 2014) explaining the significant difference found in the diversity pattern of *Mentha* and *Artemisia* as compared to maize and other MAPs included in the study.

### Structural Changes in Bulk and Rhizospheric Microbial Communities

Species diversity is a community parameter, which is related to the degree of stability of that community (Bardgett et al., 2014). In the rhizosphere, the plant selects those microbes that can utilize the nutrients efficiently from total microbial diversity (Hartmann et al., 2009; Berendsen et al., 2012), thereby creating a selection pressure. The higher number of OTUs in the maize rhizosphere was because of the presence of high amounts of simple sugars in the root exudates that attracted bacterial and fungal communities in a manner similar to the establishment of mycorrhizal associations (Broeckling et al., 2008; Berg and Smalla, 2009).

In bulk soil of both *Mentha* and *Artemisia*, the presence of leaf fall with antimicrobial activity caused a reduction in the number of OTUs as compared to rhizospheric soil. *Mentha* was less severely affected probably because it is a short-duration (3–4 months) crop and the leaf fall with antimicrobial essential oil is volatile in nature, whereas *Artemisia* is a long-duration crop with continuous leaf fall continuously leaving artemisinin into the soil, which is not volatile and less degradable as compared to the *Mentha* essential oil. As the leaf fall covers more of bulk soil, artemisinin content was found to be higher in bulk soil, so the results are more marked in AB than in MB.

### Diversity Shift in Bacterial and Fungal Communities

Bacterial commune responded less severely than the fungal commune to the presence of bioactive compounds in the soil. The unicellular and smaller size of bacteria make them exposed only to their immediate surroundings, i.e., microniche (Vos et al., 2013) with very specific conditions separating them from the direct influence of plant root, and this could be the reason why bacterial communities are not significantly different under different plants (Urbanova et al., 2011). In the case of fungi, the root-associated mycelia extending from the rhizosphere to the bulk makes them more sensitive to plantation type and other factors (Urbanova and Baldrian, 2015). Similar observations were made in our study where bacterial OTUs were not considerably affected in *Mentha* and *Artemisia*, whereas the decrease in fungal OTUs was drastic.

In case of *Mentha*, high leaf fall at the time of maturity and their decomposition probably improved the growth of degrading bacteria, which may attract diverse communities capable of utilizing sugars (Jiang et al., 2017) as well as secondary metabolites, making it more diverse and richer than rhizospheric bacterial community as observed in MB soil. The outcome of these complex processes is the development of a rhizospheric microbial community that differs noticeably from the source communities of bulk soil (Fan et al., 2017). However, the above condition reversed in AB soil, where considerable defoliation occurred in the bulk soil increasing the signatures of secondary metabolites with little input of sugar source, decreasing both bacterial and fungal community richness/diversity.

At the phylum level, known bacterial phyla were detected, which accounted for 99.5%, while the unknown ones occupied only about 0.5% of the total population. Among all the phyla identified, the members of Proteobacteria, Actinobacteria, Firmicutes, and Acidobacteria were highly abundant in all the soils. Proteobacteria is a functionally diverse group of fast-growing Gram-negative bacteria, especially plant growth promoters (Gupta, 2000) and possess the ability to utilize a broad range of root-derived carbon substances (Philippot et al., 2013a, b), making their population highest in each soil type. Actinobacteria have an important role in the decomposition of organic material (Baldrian et al., 2012) and thus play an important role in organic matter turnover and nutrient cycling. Increased abundance of Actinobacteria in MB soil may replace or reduce the abundance of other microbes, including those found useful in improving productivity and reducing the population of phytopathogens. Firmicutes are spore-forming phyla that live in the soil in small pockets of anaerobic habitats created by them, thereby reducing the effect of secondary metabolite (fallen litter of *Mentha*) firmicutes, making them highly abundant in MB soil samples. Members of phylum Acidobacteria are oligotrophic and enriched in soils with very low resource availability, whereas Chloroflexi is an anaerobic heterotroph that plays an important role in litter decomposition (Purahong et al., 2016). Acidobacteria and Chloroflexi are slow-growing entities (Jones et al., 2009) but were substantially higher in abundance in MB and AR, probably because of the sufficient amount of litter availability and nature of root exudates, respectively. Besides this, a higher abundance of Cyanobacteria, Planctomycetes, Bacteroidetes, and Verrucomicrobia were found in rhizospheric soil. All the phyla members decreased drastically in both bulk and rhizospheric soil of *Artemisia* except Chloroflexi and Cyanobacteria, which were found to be higher in AR as compared to CR. In all the cases, bacterial diversity was higher than fungal diversity, and bacterial communities were more even.

In the case of a fungal community, at the phylum level, Ascomycota and Basidiomycota are known to produce various organic matter digestive enzymes, thereby occupying the highest abundance. Glomeromycota forms arbuscular mycorrhizal association with roots of higher plants, so they were found to be higher in rhizosphere as compared to bulk soil of *Mentha* and *Artemisia*. Members of Zygomycota and Chytridiomycota (known to utilize dead plant material) were higher in bulk soil, as bulk soil contained more organic matter due to high litterfall, which served as an effective nutrient source for them. More unidentified phyla were found in bulk soil in comparison to rhizospheric soil samples in all the plant type. This suggests that although a vast number of microbes have been identified, there is still a significant proportion of unidentified microbial genera (Uroz et al., 2010).

### Use of Intercropping and Organics in Mitigating the Diversity Loss (State Shift vs. Community Rescue)

Biodiversity includes species richness (the number of species) and evenness (how well distributed abundance or biomass among species), which are considered among the most important measures (Wittebolle et al., 2009). The community evenness confers the functional stability of an ecosystem and highly uneven communities or extreme dominance of one or a few species results in an ecosystem vulnerable to environmental stress (Chapin et al., 2000; Wittebolle et al., 2009). In our study, higher evenness in MA indicates the existence of stable microbial community, which may provide functional stability during environmental fluctuations (Balvanera et al., 2005; Awasthi et al., 2014).

Monocropping damages the soil ecology and crop productivity by depleting the soil nutrient, resulting in an unbuffered niche (Marais et al., 2012). The same crop when grown incessantly causes a continuous loss of the similar type of nutrients, creating a nutrient shock for the microbial community, thereby decreasing the soil microbial diversity and increasing the abundance of only some specific candidates (Marais et al., 2012). In the case of *Mentha*, a considerable leaf fall of essential oil-bearing leaves could have greatly affected the microbial diversity because of the antimicrobial nature of these oils. This was clearly reflected in our experiments where the soil that was continuously cropped with *Mentha* had low microbial diversity. The other objective of the study was to look at the ways that could restore the microbial diversity losses by the introduction of some organic/green manures and selected crops which have shown some promise in improving microbial diversity and abundance in our preliminary studies (unpublished data). *Sesbania*, a nitrogen fixer (Dreyfus and Dommergues, 1980), may have supported the abundance of phosphate solubilizers secreting higher amounts of organic acids, lowering the soil pH, and thereby influencing the microbial diversity. As green manure, *Sesbania* might have increased the organic matter of soil supporting the microbial growth and increasing microbial diversity as reflected in our study. In our experiment, higher numbers of OTUs were observed in M_S, and M + VC cultivated soil as compared to other plants. *Chlorophytum borivilianum* (safed musli), an eminent medicinal plant, also improved the evenness of the microbial community, which could be functionally more efficient, thereby playing a role in enhancing productivity. Amendment of organics (VC/FYM) helps the soil to mitigate the imbalance caused by monocropping (Xiong et al., 2015). The higher water holding capacity and organic matter content of VC makes it highly suitable for replenishment of microbial diversity (Lim et al., 2015). It indicates that after the leaf fall of *Mentha* in the soil (which starts after about 70 days), secondary metabolite/essential oils are immediately released to the soil environment because of the presence of oil glands on the surface of the leaves. These essential oils may cause a drastic reduction in the microbial richness including degraders initially, which may cause a nutrient shock for the microbes. On the other hand, the presence of organic matter and abundant nutrients in VC may alleviate microbes from such stress. Later, with the loss of volatile essential oils from the soil, there could be a revival of many degraders, which may start the degradation of defoliated leaves and provide nutrients to the plants. Though samples were not collected at the initiation of leaf fall in *Mentha*, it is assumed that the loss of diversity could be much higher at that time than at the time of harvesting, which occurs after 90–100 days. Our results suggest that the effect of MAPs cultivation on soil microbial diversity is difficult to generalize under negative or positive impacts. However, it induces the shifts in microbial community structure, which may affect soil health and productivity. Some communities, by using organic and green manure as well as other MAPs, rescue itself or resilience takes place, which will be beneficial for soil health. Higher abundance of decomposers and growth-promoting microbes may minimize the effect of essential oil-bearing leaves by quickly decomposing the leaves aiding in faster volatilization of oil, and the resultant manure may support the rest of the microbiome to colonize efficiently there.

## Conclusion

These essential oil-bearing crops, especially *Mentha*, are gaining popularity because of their potential to generate higher incomes and they fit well into the cropping systems. We presumed that the continuous incorporation of defoliated essential oil-bearing leaves into the soil might affect the microbial diversity, resulting in the loss of productivity. Studies conducted in our laboratory indicated that the microbial diversity was low in fields continuously cropped with *Mentha* for 4 years, with the effects being more pronounced in the case of the fungal community. Efforts were made to reduce the loss in microbial diversity using various organic/green manures and some rotational crops, and it was observed that the use of organics and green manure could not only prevent the loss and richness of microbial community but also maintain the evenness that promotes the ecosystem function and improves fertility as well as productivity of the soil. This was also demonstrated in pot experiments where supplementation of VC and inclusion of *Sesbania* as a rotational crop could negate the adverse effects of loss in diversity and thereby improves the yields of *Mentha* even after continuous cropping.

## Data Availability Statement

The datasets generated for this study can be found in NCBI Sequence Read Archive (SRA) database, SUB2488937, SUB2604678, SUB2604678, SUB2659621, SRP150923, and SRP150978.

## Author Contributions

AK had the original idea for the experiments. PM and AA conducted the laboratory work. PM organized and performed the experimental design and statistical analyses on the basis of raw data provided by Genotypic and Bioinvid. PM and DM principally wrote the manuscript, with extensive input from AK, CB, and DS. All authors contributed to the ideas and writing of the manuscript.

## Conflict of Interest

The authors declare that the research was conducted in the absence of any commercial or financial relationships that could be construed as a potential conflict of interest.

## Footnotes

1 http://trex.biohpc.org/
