# Supplementary material for: Vulnerability of Soil Microbiome to Monocropping of Medicinal and Aromatic Plants and Its Restoration Through Intercropping and Organic Amendments
Source: Front Microbiol. 2019 Nov 19;10:2604. doi: 10.3389/fmicb.2019.02604 (PMC6877478; doi:10.3389/fmicb.2019.02604)
Supplement: Supplementary file 1 [file Table_1.DOCX]

**Supplementary**

**Next Generation Sequencing**

***Methodology and Quality Check of PCR for V3-V4 region / ITS region***

***Materials***

Ex Taq (TaKaRa, Code # RR001A), 2x KAPA HiFiHotStartReadyMix (KAPA Biosystems # KM2602), In-house V3-V4 regions primers for Metagenome, In-house ITS regions primers for Metagenome, 50X TAE buffer (MP, #TAE50X01), 100bp Ladder (NEB, Cat #N3231S), Agarose

***Methods***

DNA was extracted using the Power Soil DNA Extraction Kit (Molbio Laboratories), following the manufacturer’s instructions. PCR Quality Control was carried out for 6 samples of Metagenome for V3 and V4 region of 16s coding DNA sequence and ITS region of 18s coding DNA sequence at the Genotypic Technology’s Genomics facility. The DNA samples were quantified using NanoDrop. 25ng of DNA was taken as input for PCR reaction. Then PCR reactions were set up for all samples, with 1 non – template control and 1 positive control. The amplicons of the desired size were validated by running on 2% Agarose Gel. All samples showing band at expected size ~550 bp for V3-V4 region and ~750bp for ITS 1 and ITS 2.The samples were processed for library preparation.

***Library Preparation for 16s V3-V4 /ITS region***

***Materials***

TaKaRa Ex Taq™ (TaKaRa, #RR001), HighPrep PCR (Magbio, Cat # AC-60050), Nuclease Free Water (Ambion, Cat #AM9938), High Sensitivity Bioanalyzer Kit (Agilent, Cat # 5067-4626), Qubit DNA HS kit (Invitrogen #863180), Nextera XT index kit v2 (Illumina, #FC-131-2002), 100 bp ladder (NEB, #N3231), 10X TAE buffer (Invitrogen, #15558026).

***Methods***

Library preparation was performed at Genotypic Technology’s Genomics facility. 25-100ng of nanodrop quantified DNA was used for amplifying V3-V4 region of 16S region and ITS1+ITS2 combined region of 18S with specific primers which also have a ‘tag’ sequence that is complementary to Illumina sequence adapter and index primers from the Nextera XT Index kit V2. This round of PCR generates single Amplicon of ~550 bp for 16s V3-V4 regions and 740-750 bp for ITS1+ITS2 for 18s regions. The Amplified products were checked on the Agarose Gel before proceeding for Indexing PCR. In the next round of PCR (indexing PCR) Illumina sequencing adapters and dual Indexing barcodes are added using limited cycle PCR to give a final product of ~600 bp for 16s V3-V4 regions and 840-850bp for ITS1+ITS2 for 18s regions. The library was cleaned using HighPrep PCR (Magbio, Cat # AC-60050) magnetic beads and was Qubit quantified and validated for quality by running an aliquot on High Sensitivity Bioanalyzer Chip (Agilent). The following adapter sequences were used for 16s V3-V4 region.

AATGATACGGCGACCACCGAGATCTACAC [i5] TCGTCGGCAGCGTC, and

CAAGCAGAAGACGGCATACGAGAT [i7] GTCTCGTGGGCTCGG and the adapter sequences used for ITS1-ITS2 of 18s regions were AATGATACGGCGACCACCGAGATCTACAC [i5] TCGTCGGCAGCGTC, and CAAGCAGAAGACGGCATACGAGAT [i7] GTCTCGTGGGCTCGG. All the libraries show an expected size of ~600 bp for V3-V4 region with an effective insert size of ~460 bp flanked on each size by adapters with a combined size of ~140bp, while for ITS regions an expected size of ~830-850bp for ITS 1 and 2 combined region with an effective insert size of ~690-710 bp flanked on each side by adapters with a combined size of ~140bp were obtained. The libraries are suitable for sequencing on Illumina platform.

***Methodology for 16s /ITS Analysis***

Demultiplexing was done using bcl2fastq v1.8.3 conversion software. The Illumina paired-end raw data was quality checked using FastQC1. The raw reads with adapter sequences and low-quality bases were removed using an automated perl code. The processed high quality reads with more than 70% bases having Phred score greater than 30 were considered significant for further downstream analysis. The dataset was analyzed using Qiime2 v 1.9.0 with default parameters by using Greengenes3 v 13.8 database at the backend. We used closed-reference OTU picking method in this study, where the reads were clustered against a reference sequence collection and any reads which do not hit a sequence in the reference sequence collection were excluded from downstream analysis. OTUs were clustered to represent 97% similarity. This provided information on the microbial lineages found in microbial samples. The dataset was clustered at 97% similarity and mapped to the reference using UCLUST ref method. Taxonomic annotation from kingdom to species level was ascertained based on the hits with 97% similarity. Abundance graphs were plotted based on the number of hits. Comparative taxa summary plots and Heatmap was generated across the samples at phyla level considering relative abundance values using a cut-off greater than 0.1%. Krona charts were plotted using Krona5 tools for each sample. It is an interactive plot that displays the quantitative phylogenetic tree. Pie-charts were generated for each sample from phylum to species with a cut-off of 0.5% based on the absolute abundance count.

Alpha diversity was calculated using various matrices such as Chao1, observed OTUs, Shannon, and Simpson index. It reflects the diversity within the sample based on the abundance of various OTUs within a community. Rarefaction curve was plotted using QIIME based on certain rarefaction depth. The plot shows the annotated species richness in V3-V4 samples. This curve is a plot of the total number of observed OTUs annotated as a function of the number of sequences sampled. On the left, a steep slope indicates that a large fraction of the species diversity remains to be discovered. If the curve becomes flattered to the right, a reasonable number of individuals have been sampled: more intensive sampling is likely to yield only a few additional species. Sampling curves generally rise very quickly at first and then level off towards an asymptote as fewer new OUT’s are found per unit of individuals collected.


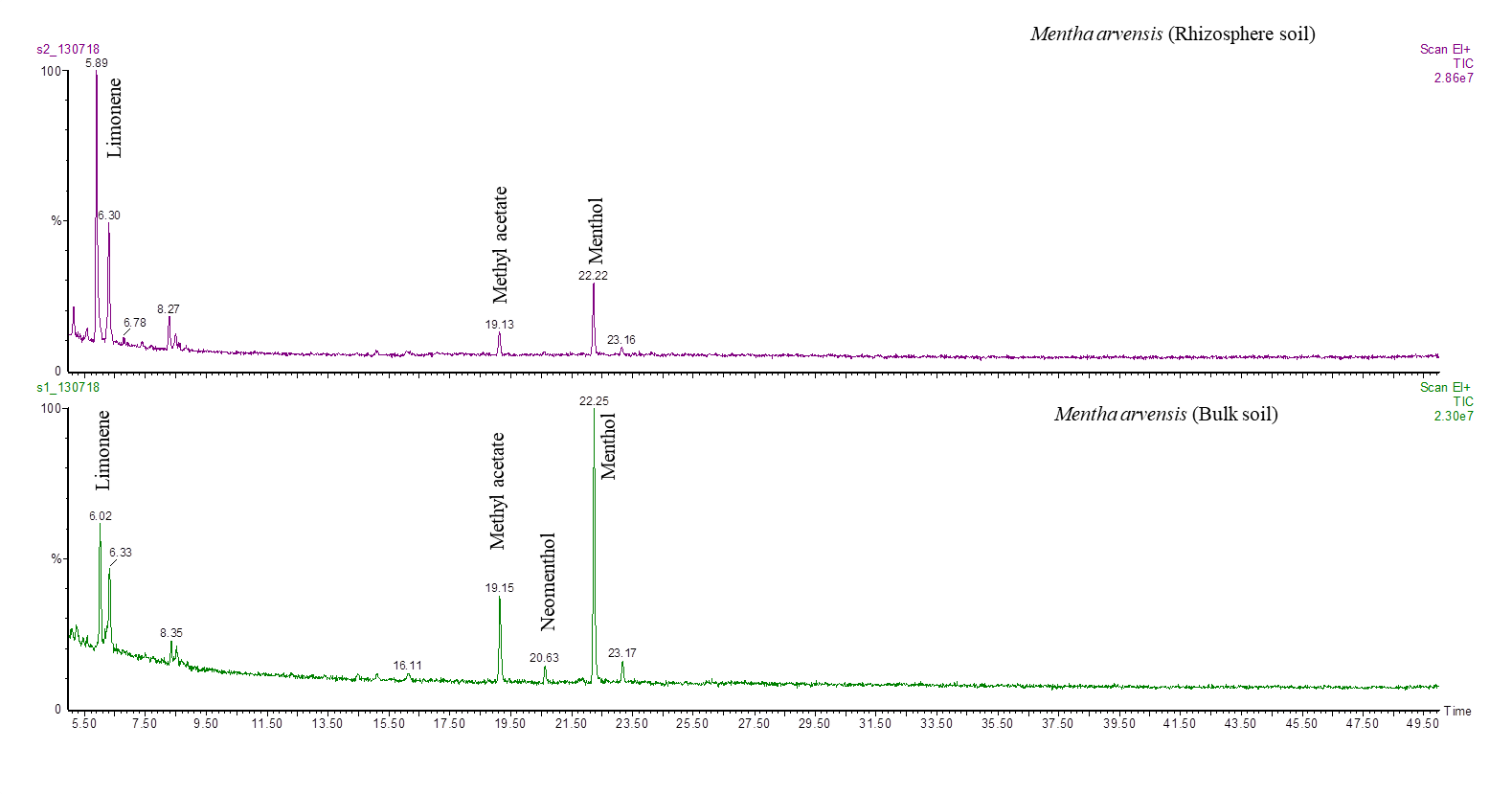


Fig S1 (a): GC/MS analysis of *Mentha arvensis* rhizospheric and bulk soil samples


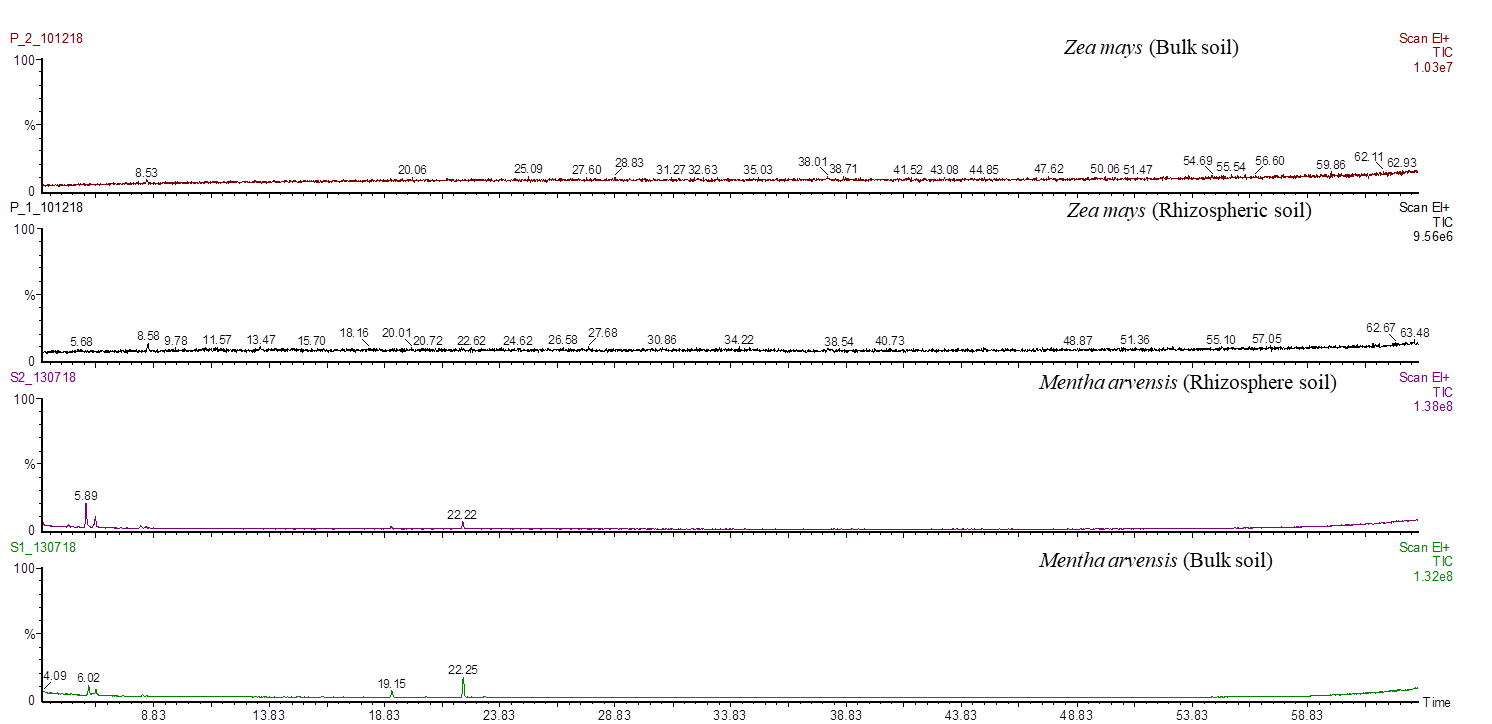


Fig S1 (b): GC/MS analysis of *Mentha* compared with control (*Zea* *mays*)


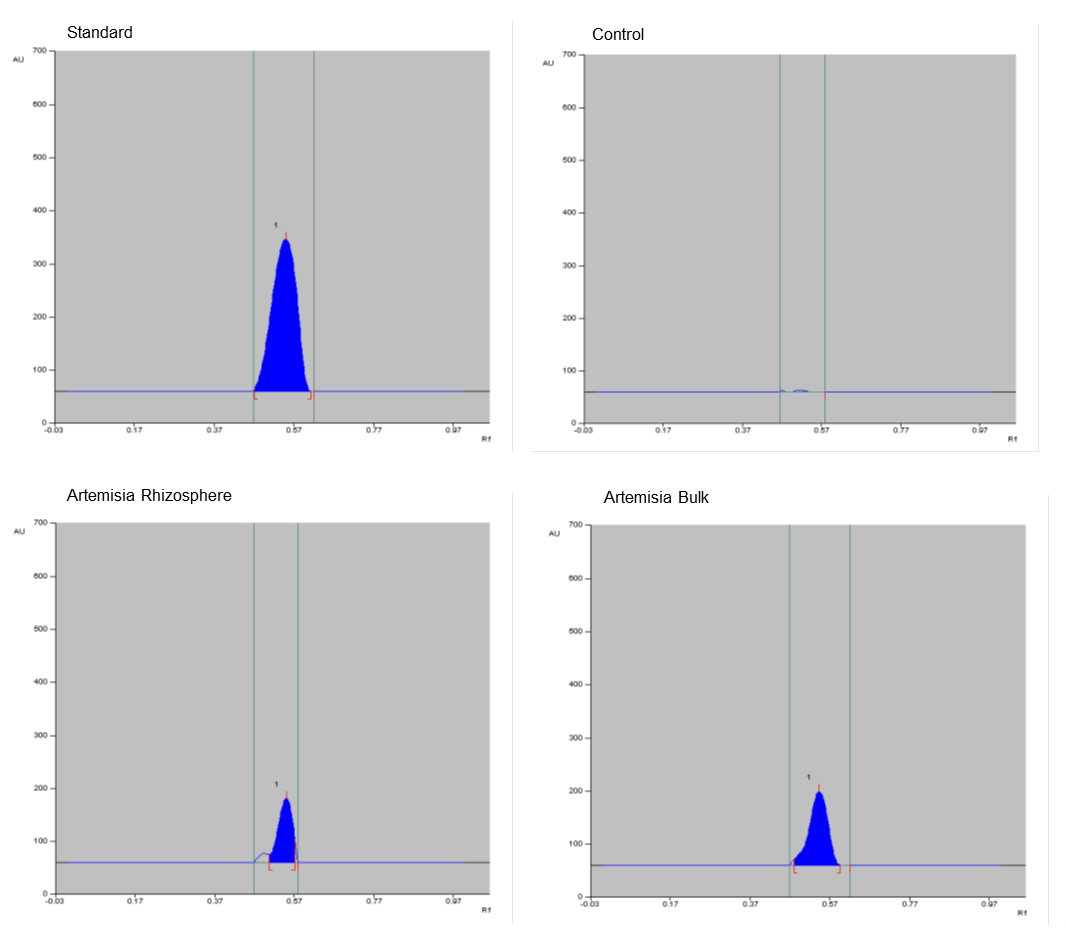


Fig S1(c): HPTLC chromatogram of artemisinin content of *Artemisia* *annua* rhizospheric and bulk soil compared with control and standard.


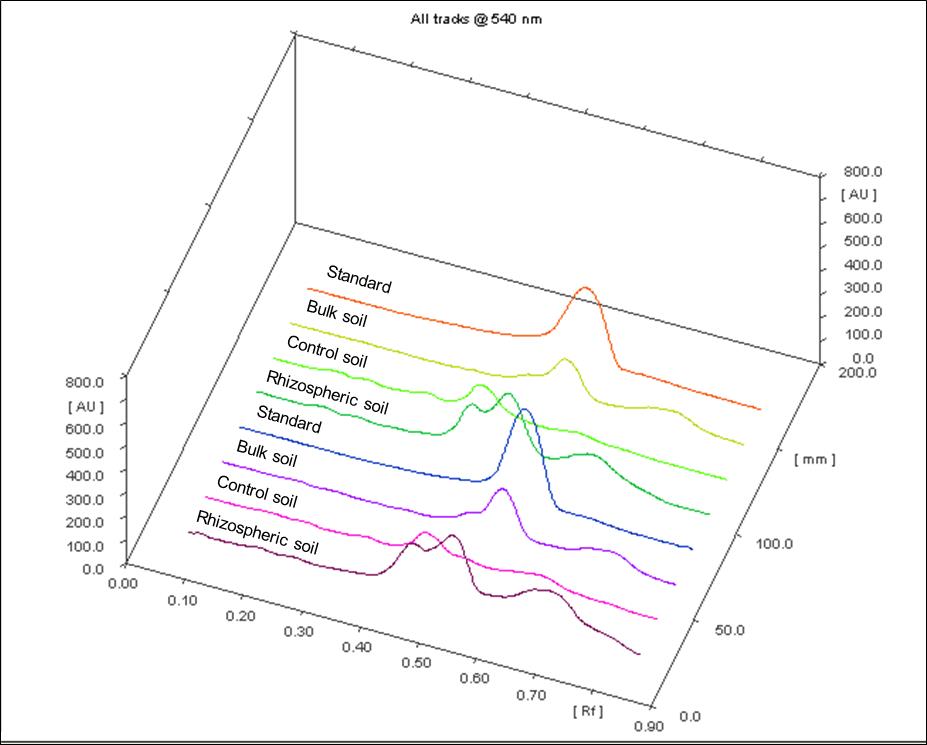


Fig S1(d): HPTLC chromatogram artemisinin content of *Artemisia* *annua* rhizospheric and bulk soil compared with control and standard.


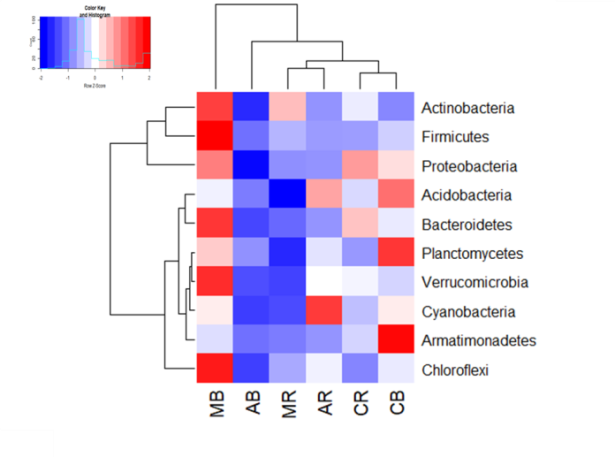


1. (c)


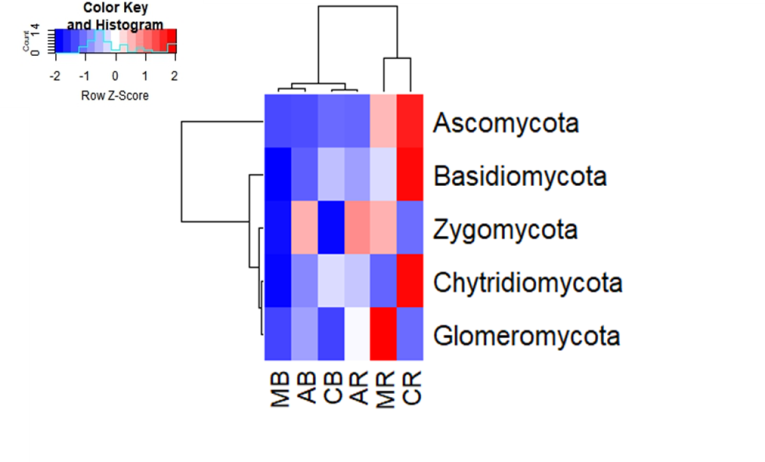


1. (d)

Fig S2a and b Abundance and c and d Heatmap of dominant phyla of both bacterial and fungal community. AB= *Artemisia* Bulk, AR= *Artemisia* Rhizosphere, MB=*Mentha* Bulk, MR=*Mentha* Rhizosphere, CB=Control Bulk, CR=Control Rhizosphere.

Bacterial community


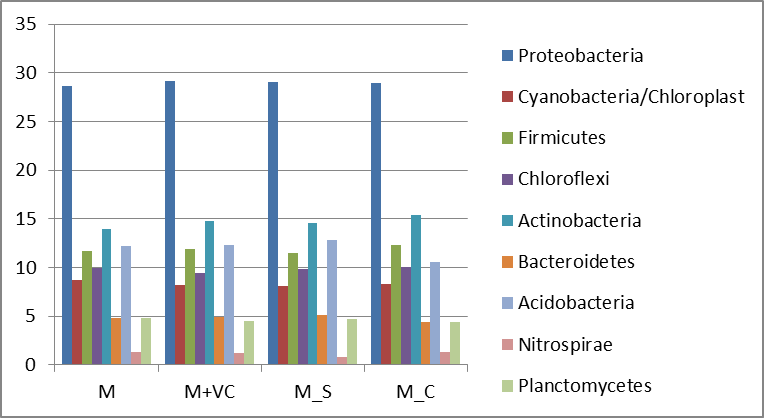


**Bacterial Community**

(a)

(b)

Fig S3a and b: Abundance of dominant bacterial and fungal community at phyla level of soil samples of Second plantation. M=*Mentha*, M+VC=*Mentha* with Vermicompost, M_S=*Mentha* before *Sesbania*, M_C=*Mentha* before *Chlorophytum*.

**Table S1 Diversity indices of field experiment of various MAPs**

| **S.N.** | **Soil samples** | **Texa** | | **Simpson_1-D** | | **Shannon_H** | | **Margalef** | | **Fisher_alpha** | |
| --- | --- | --- | --- | --- | --- | --- | --- | --- | --- | --- | --- |
|  |  | **16S** | **ITS** | **16S** | **ITS** | **16S** | **ITS** | **16S** | **ITS** | **16S** | **ITS** |
| 1 | MAP1 | 77 | 44 | 0.8894 | 0.8431 | 3.079 | 2.44 | 14.34 | 8.116 | 45.85 | 17.44 |
| 2 | MAP2 | 52 | 23 | 0.8763 | 0.7983 | 2.801 | 2.139 | 9.626 | 4.152 | 22.82 | 6.71 |
| 3 | MAP3 | 49 | 23 | 0.834 | 0.7544 | 2.609 | 1.952 | 9.059 | 4.152 | 20.7 | 6.71 |
| 4 | MAP4 | 60 | 23 | 0.8359 | 0.8032 | 2.582 | 2.079 | 11.14 | 4.152 | 29.06 | 6.71 |
| 5 | MAP5 | 52 | 30 | 0.827 | 0.7964 | 2.527 | 2.283 | 9.62 | 5.473 | 22.82 | 9.788 |
| 6 | MAP6 | 89 | 53 | 0.8523 | 0.8984 | 2.801 | 3.013 | 16.61 | 9.814 | 61.48 | 23.55 |
| 7 | MAP7 | 67 | 42 | 0.8547 | 0.9075 | 2.691 | 3.043 | 12.46 | 7.738 | 35.34 | 16.21 |
| 8 | MAP8 | 32 | 22 | 0.8495 | 0.6967 | 2.321 | 1.735 | 5.85 | 3.963 | 10.76 | 6.308 |
| 9 | MAP9 | 25 | 21 | 0.772 | 0.7747 | 2.05 | 1.992 | 4.53 | 3.775 | 7.542 | 5.916 |

MAP1=Control, MAP2=*Bacopa monnieri* (Bacopa), MAP3=*Andrographis paniculata* (Kalmegh), MAP4=*Ocimum basilicum* (Basil), MAP5= *Cymbopogon citratus* (Lemon grass), MAP6=*Chrysopogon zizanioides* (Vetiver), MAP7=*Palargonium graveolens* (Geranium), MAP8=*Artemisia annua* (Atremisia), MAP9=*Mentha arvensis* (Mint)

**Table S2. HPTLC of Artemisia grown field soil (rhizosphere and bulk soil)**

| **S.N.** | **Soil samples** | **Artemisinin content (ppm)** |
| --- | --- | --- |
| **1.** | **AR** | 0.16±0.004 |
| **2.** | **AB** | 0.25±0.01 |
| **3.** | **CB** | BDL |
| **4.** | **CR** | BDL |

Data are expressed as average with standard deviations (n = 3). AR= *Artemisia* Rhizosphere , AB= *Artemisia* Bulk,

CB= Control Bulk, CR= Control Rhizosphere

**Table S3. Physicochemical parameter of MA, AA and control (bulk and rhizosphere) field soil samples**

| **S.N.** | **Soil Samples** | **pH** | **EC(µs/cm)** | **N(kg/H)** | **P(kg/H)** | **K(kg/H)** | **OC(%)** |
| --- | --- | --- | --- | --- | --- | --- | --- |
| **1.** | **AB** | 6.9±0.09d | 59.4±1.9c | 163.3±1.5e | 11.6±0.5c | 188.6±1.79b | 1.31±0.002a |
| **2.** | **AR** | 8.3±0.09a | 183.3±25.1b | 222.3±0.8b | 20.7±1.4b | 198.2±0.043a | 132±0.001a |
| **3.** | **MB** | 7.25±0.04c | 69.6±0.8c | 175.8±1.2d | 12.5±0.6c | 203.77±1.12a | 1.32±0.002a |
| **4.** | **MR** | 8.17±0.05a | 227.0±2.1a | 232.2±1.5a | 22.9±1.4b | 199.16±0.48a | 1.32±0.002a |
| **5.** | **CB** | 7.8±0.08b | 70.4±1.1c | 188.3±1.9c | 21.6±0.5b | 165.05±2.30c | 1.27±0.01b |
| **6.** | **CR** | 8.3±0.09b | 64.2±1.4c | 225.4±2.3b | 32.3±1.9a | 163.47±1.51c | 1.20±0.01c |

Different letters show significant difference at P = 0.05. Data are expressed as average with standard deviations

(n = 3). AB= *Artemisia annua* bulk soil, AR= *Artemisia annua* rhizospheric soil, MB=*Mentha arvensis* bulk soil, MR=

*Mentha arvensis* rhizospheric soil, CB= Control Bulk soil, CR=Control rhizospheric soil.

**Experimental design to investigate the role of organic manures and crop plants for restoring the loss in microbial diversity**

| **1^st^ plantation** | **2^nd^ plantation** | **3^rd^ plantation** |
| --- | --- | --- |
| Blank (B) | Blank (B) | Blank (B) |
| Mentha(M) | Mentha(M) | Mentha(M) |
| Mentha+VC  (M+VC) | Mentha+VC  (M+VC) | Mentha+VC  (M+VC) |
| Mentha+FYM  (M+FYM) | Mentha+FYM  (M+FYM) | Mentha+FYM  (M+FYM) |
| Mentha  (M) | Sesbania  (M_S) | Mentha  (M_S_M) |
| Mentha  (M) | Trigonella  (M_T) | Mentha  M_T_M |
| Mentha  (M) | Chlorophytum  (M_C) | Mentha  M_C_M |
| Mentha  (M) | Ocimum  (M_O) | Mentha  M_O_M |

**Table S4. Physicochemical parameters of microbial diversity restoration experiment of MA.**

| **S.N** | **Soil samples** | **pH** | **EC(µs/cm)** | **N(kg/H)** | **P(kg/H)** | **K(kg/H)** | | **OC(%)** | |
| --- | --- | --- | --- | --- | --- | --- | --- | --- | --- |
|  | **After first plantation** | | | | | | | | |
| **1.** | **B** | 8.1±0.13a | 205.9±10.56b | 206.3±11.05b | 32.53±7.87d | 164.89±12.0b | | 1.23±0.02a | |
| **2.** | **M** | 8.63±0.27a | 236.4±11.25a | 235.41±10.9a | 79.45±4.47b | 154.39±13.8b | | 1.49±0.07a | |
| **3.** | **M+VC** | 8.22±0.8a | 208.8±12.01b | 227.75±12.15ab | 54.59±7.39c | 161.4±12.79b | | 1.37±0.09a | |
| **4.** | **M+FYM** | 8.4±0.23a | 225.1±14.4ab | 216.73±13.03ab | 97.14±5.8a | 240.35±16.0a | | 1.24±0.15a | |
|  | **After second plantation** | | | | | | | | |
| **1.** | **B** | 8.64±0.2a | 436±11.8a | 231.6±14.7c | 81.1±4.01a | 151.9±11.1d | | 1.39±0.4a | |
| **2.** | **M** | 8.2±0.1b | 355.3±10.3b | 208.5±10.5d | 43.1±6.2c | 174.7±10.9bc | | 1.42±0.1a | |
| **3.** | **M+VC** | 8.39±0.16ab | 366±14.0b | 204.7±11.3d | 46.9±7.4bc | 130.7±10.7e | | 1.54±0.4a | |
| **4.** | **M+FYM** | 8.23±0.19b | 294.3±11.1de | 224.9±12.6cd | 56.7±5.1b | 204.4±14.3a | | 1.3±0.08a | |
| **5.** | **M_S** | 8.17±0.1b | 313±12.2cd | 265±13.9a | 32.3±4.7d | 165.3±10.9cd | | 1.6±0.6a | |
| **6.** | **M_T** | 8.21±0.17b | 317.4±14.8c | 212.6±10.2cd | 44.3±4.1c | 197.4±13.3ab | | 1.2±0.09a | |
| **7.** | **M_C** | 8.04±0.2b | 324.7±13.1c | 264.9±11.4b | 53.6±7.1bc | 163.2±11.7cd | | 1.31±0.3a | |
| **8.** | **M_O** | 8.15±0.3b | 285.3±11.7e | 235.4±12.4c | 33.0±4.4d | 206.8±12.2a | | 1.63±0.07a | |
|  | **After third plantation** | | | | | | | | |
| **1.** | **B** | 8.2±0.1bc | 225.7±10.0e | 211.8±16.8c | 43.0±6.15de | | 174.8±12.2b | 1.17±0.18d |  |
| **2.** | **M** | 8.13±0.1c | 225.6±12.2e | 264.1±13.6a | 52.7±4.85c | | 162.2±11.6bc | 1.52±0.2ab |  |
| **3.** | **M+VC** | 8.53±0.1a | 273.2±11.7d | 239.3±15.7b | 80.7±5.57b | | 153.4±13.7bcd | 1.43±0.16cd |  |
| **4.** | **M+FYM** | 8.28±0.17abc | 314.1±13.8bc | 216.9±12.4bc | 97.3±4.01a | | 243.6±15.2a | 1.62±0.1ab |  |
| **5.** | **M_S_M** | 8.45±0.14ab | 320.7±14.6b | 205.9±14.3c | 48.3±3.18cd | | 130.3±10.2d | 1.71±0.3a |  |
| **6.** | **M_T_M** | 8.27±0.10bc | 294.2±12.8cd | 172.7±11.6d | 35.1±4.64ef | | 159.9±17.3bc | 1.17±0.08d |  |
| **7.** | **M_C_M** | 8.19±0.16bc | 324.9±11.2b | 263.7±10.01a | 33.1±5.14f | | 165.8±11.9bc | 1.21±0.06d |  |
| **8.** | **M_O_M** | 8.3±0.17abc | 359.1±15.6a | 226.8±11.6bc | 47.1±2.6cd | | 140±14.9cd | 1.25±0.08cd |  |

B= Blank, M=*Mentha*, VC=vermicompost, FYM=farm yard manure, S=*Sesbania*, T=*Trigonella*, C=*Chlorophytum*, O=*Ocimum*. Different letters show significant difference at P = 0.05. Data are expressed as average with standard deviations (n = 3).

**Table S5 Diversity indices of restoration experiment of MA**

| **S.N** | **Soil samples** | **Taxa** | | **Simpson** | | | **Shannon** | | **Marglef** | | | | | **Fisher’s α** | | | | | |
| --- | --- | --- | --- | --- | --- | --- | --- | --- | --- | --- | --- | --- | --- | --- | --- | --- | --- | --- | --- |
|  |  | **16S** | **ITS** | **16S** | | **ITS** | **16S** | **ITS** | **16S** | | **ITS** | | | **16S** | | | | | **ITS** |
|  | **After first harvesting** | | | | | | | | | | | | | | | | | | |
| **1.** | **B** | 21 | 23 | 0.02261 | | 0.1876 | 0.7739 | 0.8124 | 1.893 | | 2.134 | | | 0.3162 | | | | | 0.3521 |
| **2.** | **M** | 21 | 24 | 0.7747 | | 0.8319 | 1.992 | 2.249 | 3.775 | | 4.341 | | | 5.916 | | | | | 7.121 |
| **3.** | **M+VC** | 42 | 25 | 0.9075 | | 0.7175 | 3.043 | 2.163 | 7.738 | | 5.211 | | | 16.21 | | | | | 10.7 |
| **4.** | **M+FYM** | 30 | 23 | 0.835 | | 0.815 | 2.433 | 2.153 | 5.473 | | 4.152 | | | 9.788 | | | | | 6.71 |
|  | **After second harvesting** | | | | | | | | | | | | | | | | | | |
| **1.** | **B** | 21 | 21 | 0.8304 | | 0.7773 | 2.204 | 1.941 | 3.775 | | | 3.775 | | 5.916 | | 5.916 | | | |
| **2.** | **M** | 22 | 37 | 0.8451 | | 0.891 | 2.353 | 2.827 | 3.964 | | | 6.795 | | 6.308 | | 13.35 | | | |
| **3.** | **M+VC** | 25 | 42 | 0.8195 | | 0.9247 | 2.235 | 3.146 | 4.53 | | | 7.738 | | 7.542 | | 16.21 | | | |
| **4.** | **M+FYM** | 25 | 25 | 0.8247 | | 0.7787 | 2.302 | 2.064 | 4.53 | | | 4.53 | | 7.542 | | 7.542 | | | |
| **5.** | **M_S** | 26 | 37 | 0.83 | | 0.8273 | 2.313 | 2.474 | 4.719 | | | 6.795 | | 7.972 | | 13.35 | | | |
| **6.** | **M_T** | 24 | 29 | 0.8265 | | 0.7899 | 2.273 | 2.237 | 4.341 | | | 5.285 | | 7.121 | | 9.319 | | | |
| **7.** | **M_C** | 24 | 46 | 0.8294 | | 0.9358 | 2.238 | 3.229 | 4.341 | | | 8.493 | | 7.121 | | 18.71 | | | |
| **8.** | **M_O** | 25 | 25 | 0.8554 | 0.8001 | | 2.402 | 2.108 | 4.53 | 7.542 | | | | 4.53 | | | 7.541 | | |
|  | **After third harvesting** | | | | | | | | | | | | | | | | | | |
| **1.** | **B** | 23 | 23 | 0.783 | 0.8032 | | 1.995 | 2.079 | 4.152 | | | | 4.152 | | 6.71 | | | 6.71 | |
| **2.** | **M** | 24 | 30 | 0.822 | 0.7964 | | 2.238 | 2.283 | 4.341 | | | | 5.473 | | 7.121 | | | 9.788 | |
| **3.** | **M+VC** | 26 | 37 | 0.7915 | 0.8273 | | 2.091 | 2.474 | 4.719 | | | | 6.795 | | 7.972 | | | 13.35 | |
| **4.** | **M+FYM** | 24 | 25 | 0.8089 | 0.8001 | | 2.142 | 2.108 | 4.341 | | | | 4.53 | | 7.121 | | | 7.541 | |
| **5.** | **M_S_M** | 27 | 42 | 0.8394 | 0.9247 | | 2.382 | 3.146 | 4.907 | | | | 7.738 | | 8.412 | | | 16.21 | |
| **6.** | **M_T_M** | 25 | 37 | 0.7561 | 0.8941 | | 1.987 | 2.787 | 4.53 | | | | 6.795 | | 7.542 | | | 13.35 | |
| **7.** | **M_C_M** | 25 | 46 | 0.8311 | 0.9358 | | 2.302 | 3.229 | 4.53 | | | | 8.493 | | 7.542 | | | 18.71 | |
| **8.** | **M_O_M** | 22 | 24 | 0.7825 | 0.8129 | | 1.961 | 2.14 | 3.964 | | | | 4.341 | | 6.308 | | | 7.122 | |

B=Blank, M=*Mentha*, VC=vermicompost, FYM=farm yard manure, S=*Sesbania*, T=*Trigonella*, C=*Chlorophytum*, O=*Ocimum*.

**Table S6 Effect VC and FYM on plant biomass and oil yield of *M.arvensis* (after first harvesting)**

| **Treatment** | **Fresh weight (g)** | **Oil yield (g per pot)** |
| --- | --- | --- |
| M | 75.82 ± 8.43 b | 0.61 ±0.04 b |
| M+VC | 96.04 ± 5.33 a | 0.72 ± 0.02 a |
| M+FYM | 87.17 ± 6.53 ab | 0.62 ± 0.03 b |

M=*Mentha*, VC=vermicompost, FYM=farm yard manure. Different letters show significant difference at P = 0.05. Data are expressed as average with

standard deviations (n = 3).

**Table S7 Effect of various crops, green manure, VC and FYM on plant biomass and oil yield of *M.arvensis***

**(after third harvesting)**

| **Treatment** | **Fresh weight (g)** | **Oil yield (g per pot)** |
| --- | --- | --- |
| M | 46.81 ± 8.84 cd | 0.395 ± 0.037 cd |
| M+VC | 75.15 ± 12.43 b | 0.624 ± 0.055b |
| M+FYM | 70.83 ± 8.79 b | 0.617 ± 0.025b |
| M_S_M | 90.51 ± 16.42 a | 0.76 ± 0.042 a |
| M_T_M | 54.71 ± 5.886 c | 0.426 ± 0.039 c |
| M_C_M | 65.71 ± 5.714 b | 0.58 ± 0.0338 b |
| M_O_M | 45.25 ± 7.618 d | 0.34 ± 0.044 d |

M=*Mentha*, VC=vermicompost, FYM=farm yard manure, S=*Sesbania*, T=*Trigonella*, C=*Chlorophytum*, O=*Ocimum*. Different letters show significant difference at P = 0.05. Data are expressed as average with standard deviations (n = 3).
